# Role of botox in BPBP

**DOI:** 10.1186/1753-6561-9-S3-A17

**Published:** 2015-05-19

**Authors:** Praveen Bhardwaj

**Affiliations:** 1 Ganga Hospital, Coimbatore, 641043, India

## 

The role of Botox in Brachial Plexus Birth Palsy (BPBP) is still evolving. It is mainly used to overcome the co-contractions commonly seen in patients with recovering BPBP. Co-contractions result from cross innervation and are the main reason for development of various deformities in birth palsy patients. Unwanted muscular co-contractions or inappropriate activation of anatagonist muslces can hamper co-ordinated movements. Botox can thus help by suppressing these co-contractions. Its use has been reported for quite some time now and indications are being defined. Recent reports confirm it as an adjuvant in the treatment of posterior shoulder subluxation. What needs to be scrutinised is the exact patient group it would be helpful for. The patients who are clear candidates for surgery need not have delay in appropriate treatment due to Botox. We have been using Botox in our practice for three main indications:

## Child with limited elbow flexion

These are children who have reasonable hand function at 4-6 months but limited elbow flexion with clinical evidence of co-contraction between the biceps and the triceps. Injecting botox at this age allows the option of nerve surgery in case the recovery of elbow flexion is not satisfactory after botox in 3-4 months’ time. If the biceps is judged to be very weak or the child is older, nerve surgery is undertaken. Children with weak biceps and especially those with weak shoulder abduction are candidates for surgery and relying on Botox would only delay the surgery.

## Children with limited external rotation +/- abduction at the shoulder

Younger children (less than 1 year) who have recovered satisfactory elbow flexion and hand function but have limited shoulder external rotation +/- abduction. After Botox injection the shoulder is put in shoulder spica cast with shoulder in > 45 degree external rotation after manipulation under anaesthesia. If the child does not have adequate external rotation under anaesthesia they are unlikely to get better. We have observed better results in children who have passive external rotation of above 20 degrees under anaesthesia. Results are also better in the younger child. Early use appears to retain at least passive external rotation and probably would maintain the glenoid concentric which would make later surgical intervention more effective.

One other uncommon indication is – a child who has undergone nerve surgery to restore elbow flexion and is in the phase where the biceps has recovered but is not strong enough to bend the elbow against strong triceps. Botox paralyses triceps and unmasks the biceps function and reassures parents about the recovery of the operated muscle and with dedicated physiotherapy the biceps is made ‘stronger’ till the triceps recover.

Whatever the indication is, the safe dose of 10 units/Kg body weight is strictly followed. We avoid repeated multiple injections as the safety issue is a great concern as we are injecting functioning muscles in growing children. We have not encountered any complications related to its use.

